# Canine Lymphoma, More Than a Morphological Diagnosis: What We Have Learned about Diffuse Large B-Cell Lymphoma

**DOI:** 10.3389/fvets.2016.00077

**Published:** 2016-08-31

**Authors:** Luca Aresu

**Affiliations:** ^1^Department of Comparative Biomedicine and Food Science, University of Padova, Legnaro, Italy

**Keywords:** copy number variations, DLBCL, dog, lymphoma, methylation, transcriptome

## Abstract

Diffuse large B-cell lymphoma (DLBCL) is the most common canine aggressive B-cell lymphoma worldwide, and new recent molecular approaches have shown that DLBCL constitutes a heterogeneous tumor that cannot be unraveled by morphology and immunophenotype. DLBCL behaves aggressively, typically progressing over a short period of time, and the therapy response may be difficult to be predicted. Recently, the rate of bone marrow infiltration by flow cytometry has been demonstrated to be prognostic, but more sensible markers are needed. As the clinical behavior is different, there is vast clinical and basic research devoted to identifying prognostically or biologically distinct DLBCL subgroups. Transcriptomic analysis by gene expression profile, copy number variations by array comparative genomic hybridization and epigenetic perturbations have tentatively described this heterogeneity. Molecular subgroups using oncogenic pathways and target genes have also been correlated to different outcome in a small number of cases. The objectives of this review are to summarize the current knowledge on the biology, clinical, and pathological characteristics of canine DLBCL. To date, DLBCL probably is the most investigated tumor in veterinary medicine, and its relevance as spontaneous model for human DLBCL has been confirmed by these studies. In future, these discoveries will ultimately lead to a better understanding of the underlying disease mechanisms, possibly translating into more effective therapeutic strategies.

## Introduction

Lymphoma is a generic term that generally groups a heterogeneous disease, encompassing a number of subtypes of varying malignancy that occur as the result of neoplastic transformation of B and T lymphocytes at different stages of development. While diagnosing lymphoma as a general entity can be relatively straightforward *via* standard cytology, describing the different subtypes and anticipating their biologic behavior is still challenging (1). In addition to the morphological characteristics of neoplastic lymphocytes, the identification of the cell lineage and molecular features currently aid in the accurate classification of lymphoma. Routinely, cytology may be integrated by flow cytometry (FC). The major principles of this technique are the simultaneous measurement of a very large number of cells and the recording of multiple parameters for individual cells such as size, complexity, and fluorescence features. In contrast with other immunophenotyping methods, FC is easy to perform and can produce results in a short time. Furthermore, FC has several applications such as lineage assessment, evaluation of degree of maturation, antigen quantitation/aberration, cell proliferation activity, and tumor staging (2–6). However, the limited number of antibodies available in veterinary medicine compared with human medicine, and the risk of misdiagnosing different subtypes of lymphoma, such as indolent lymphomas, still represent the two major disadvantages of the combined use of cytology and FC (7). Another disadvantage of FC is represented by the need to obtain a sufficient number of live cells for the analysis.

Conventional histopathology is mainly associated to immunohistochemistry (IHC) providing valuable architectural and immunophenotypic information, respectively. Nowadays, the histopathologic classification proposed by the World Health Organization is recommended. An algorithm to approach the morphological diagnosis has been recently provided, and considers the following: (1) neoplastic growth pattern, (2) nuclear size, (3) nuclear morphology, (4) number of mitoses per high-power field, and (5) immunophenotype (8). Recently, a group of international veterinary pathologists achieved agreement of 80% in the application of the WHO classification criteria for the diagnosis of canine lymphoma based on histology and IHC, also highlighting the need to obtain the whole lymph node for the diagnosis, thereby discouraging the use of tru-cut biopsy. In fact, fragmented nodal tissues may be more difficult to interpret than intact organs (8). However, the invasive procedure of lymphadenectomy and its associated costs are reported as limits by practitioners.

In 2003, PCR for antigen receptor rearrangement (PARR) has been developed for dogs, and promising results have been described in the last ten years in terms of improving diagnosis and detecting minimal residual disease (9). The PARR technique refers to the concept that lymphoma is a clonal expansion of lymphocytes with specific DNA regions that are unique in both length and sequence in the neoplastic lymphocytes. An advantage of PARR is that it can be performed on cells obtained *via* aspiration, fluid samples, small tissue samples, and cell smears potentially establishing the presence of a malignancy and discriminating phenotype (9, 10). The limits of detection of neoplasia with the PCR assay depend on the sampled tissue, but the general assay is about one neoplastic cell in 1,000 non-neoplastic cells. Despite FC tends to show a higher sensitivity compared with PARR, whenever fresh samples are not available, PARR becomes fundamental for determination of immunophenotype as shown by Thalheim et al. Only recently, the use of molecular analysis to evaluate minimal residual disease in post-therapy samples has been considered to determine prognosis or opting for rescue therapy in dogs with B-cell lymphoma. However, the clinical significance of this type of testing is still controversial since consensus primers of IgH have a low sensitive for detection of minimal residual disease (11, 12). In one study, canine-specific primers have been used for residual disease detection of immunoglobulin gene rearrangements (13). Despite the method used is innovative in veterinary medicine, this is time-consuming and a quite expensive process where the original tumor clones are amplified using consensus primers, and the PCR product is then sequenced. Unfortunately, this approach is still routinely unavailable.

Even though the morphological diagnosis criteria, molecular tests, and FC performance in canine lymphoma have significantly improved in the recent years, treatment strategies have not accordingly evolved, and no therapeutic guidelines have been stratified based on the WHO histotype classification. Diffuse Large B-cell lymphoma (DLBCL) is the most frequent lymphoma histotype, and recent investigations have contributed to improve therapeutic approaches and to determine the genetic of this tumor, starting from the consideration that dogs with DLBCL have markedly different clinical courses and treatment responses. The main goal of this article is to review the most recent advances in clinical, pathologic, and molecular characteristics in dogs affected by DLBCL.

### Clinical and Prognostic Features of DLBCL

Clinically, dogs affected by DLBLC show a moderate to severe enlargement of peripheral lymph nodes (1). Histologically, DLBCL is characterized by a diffuse proliferation of large neoplastic lymphoid cells with nuclear size more than twice the size of red blood cells. The centroblast variant is the most frequent one and is characterized by a diffuse proliferation of large, not cleaved, lymphoid cells resembling the proliferating cells of the germinal center (GC) with oval to round vesicular nuclei with fine chromatin, and scanty amphophilic to basophilic cytoplasm. Nucleoli are always visible and multiple. The immunoblast variant in dogs is less frequent and considered clinically more aggressive. By IHC, DLBCLs express CD79, CD20, and the nuclear transcription factor PAX5 (14). Dogs with DLBCL have variable clinical course, response to treatment, and prognosis, which may depend on many clinicopathological and morphological parameters. Furthermore, a proportion of canine DLBCL seems to occur from evolution of follicular lymphomas and marginal lymphomas through a mechanism described as transformation. In veterinary medicine, this transformation has been characterized only by histology, but it may reflect more complicated biologic events leading to new mutations in coding regions (15).

A value of 3% infiltrating neoplastic cells in bone marrow (BM) has been defined as the most useful and feasible cut-off in dogs with DLBCL to discriminate BM infiltration. Also, lymphoma survival and time to progression were significantly associated to this value (16). Previously, Valli et al. have demonstrated that the number of mitosis is associated with clinical follow-up of dogs affected by DLBCL. Interestingly, the median survival of dogs with less than 20 mitoses per 400 field was 188 days; in contrast, the median survival of dogs with a number higher than 20 mitoses per 400 field was 31 days (14). Unfortunately, the dogs received multiple treatments and disease stages were not consistently reported.

### Molecular Pathogenesis of DLBCL

In 2013, Frantz et al. were the first authors to approach canine lymphoma by gene expression profile (GEP) analysis. Using DNA microarrays, 35 lymphomas representing the most common histologic subtypes were investigated, comprising DLBCL (17, 18). DNA microarray technology allows the screening of thousands of expressed genes in a single experiment, and the advantages of using such technique in canine lymphoma research are twice. First, transcriptome analysis of neoplastic lymphocytes compared with normal lymphocyte population may help to define the pathogenetic mechanisms underlying the tumor development. This was well demonstrated in a recent elegant paper published on canine DLBCL, where the dataset of 23 tumor samples and 10 control lymph nodes clustered in 2 well-separated clusters (19). A total of 3286 genes were differently expressed, and most of them were downregulated in DLBCL compared with normal lymphocytes (*n* = 2360). The second advantage of transcriptome analysis is the identification of specific signatures that are useful to molecularly classify the tumor that would look similar based on the morphological aspects. This type of approach was reported approximately 20 years ago in human medicine (20). A groundbreaking discovery permitted to separate human DLBCL into two distinct subtypes based on their peculiar specific gene expression signatures. One subtype was characterized by the expression of genes typical of GC B cells, the other by the expression of genes that were upregulated in activated B cells (ABC). Patients with GC-DLBCLs and ABC-DLBCLs have significantly different survival rates following chemotherapy. Whereas patients with GCB DLBCL respond favorably to rituximab and chemotherapy, more than 50% of ABC DLBCL patients will succumb to their disease (20). This pioneering study leads to the current proposal that GC-DLBCLs and ABC-DLBCLs represent two distinct biological disease entities. Similarly, in 2014, Richards et al. presented an elegant study where they applied DNA microarray technology in a group of dogs affected by B-cell lymphomas, 32 of which were diagnosed as DLBCL. Genes that distinguish ABC and GCB human DLBCL separate canine B-cell lymphomas into two groups that showed statistically different survival times (21). Finally, a list of the most significant genes were studied and were found to be involved in the same pathways and processes of human DLBCL. NF-κB signaling and B-cell receptor signaling were the most relevant ones.

### Genetic Landscape of DLBCL

Lymphoma and cancer, in general, are diseases characterized by chromosomal modifications compared with the germ line DNA, and genes that display abnormal expression in aberrant chromosomal regions are likely to be key players in tumor initiation and progression. The first reports to detect chromosomal changes in canine lymphomas were based on conventional cytogenetic (22). In recent years, the technology of the array comparative genomic hybridization (aCGH) has been developed also in veterinary medicine giving several advantages. The primary advantage of aCGH over fluorescence *in situ* hybridization is the array’s ability to detect DNA copy changes at multiple loci in a genome within a single experiment, including deletions, duplications, or amplifications. The aCGH method uses slides arrayed with small segments of DNA as target for analysis. Instead of using metaphase chromosomes, the array is created by the deposit and immobilization of small amounts of DNA, called probes, on a solid support. Two studies using oligonucleotide aCGH have been performed in canine lymphomas, which result in a gross overview of chromosomal imbalance patterns in this tumor (23, 24).

To elucidate copy number variations (CNVs) in cDLBCL, we analyzed and compared by aCGH technique, a set of 12 dogs with naive DLBCL and treated with similar protocols (17). Relative skin punch biopsies were used as DNA reference. The total pattern of genomic aberrations consisted of 90 different genomic imbalances (mean per tumor, 17). The first aim of the study was to identify genomic regions or even CNVs associated with the clinical course of the disease. Interestingly, a gain of chr13 was significantly more frequent in dogs that presented a complete response after the end of treatment compared with dogs that relapsed during treatment and in dogs initially diagnosed with stage III and IV disease. On the contrary, the gain of the entire chr31 was more frequent in dogs with more severe prognosis. Furthermore, since chemotherapy is known to be a possible accelerator of clonal evolution, and different patterns of repopulation post therapy have been identified in human cancers, going from stable equilibrium of subpopulations to alternated dominance between subclones over time, we compared ACGH profiles in a subset of dogs that relapsed during their lifetime. Interestingly, these dogs showed a lower number of genomic imbalances (mean per tumor, 10; range, 7–14), equally distributed in gains and losses. In all cases, at least one common genomic abnormality was found both in the primary DLBCL and relative relapse, confirming the clonal relationship between matched tumors (24).

Unfortunately, no works have been performed until now integrating GEP and copy number data sets in canine cancer. Combining analysis of DNA copy number and gene expression microarrays of the same tumor samples has revealed, in human tumors, variable direct effect of allelic imbalance on gene expression. Acquisition of both gene copy number and expression data for the same set of samples presents an opportunity to ask how gene copy number influences mRNA levels. Significant data are expected in the future to corroborate and to combine results that have been published so far in canine lymphoma.

### Alterations in DNA Methylation in DLBCL

A last topic in canine lymphoma research is related to a promising concept that has been recently developed in different human lymphoproliferative diseases where aberrant DNA methylation has been demonstrated to play a key role in hematopoietic tumor development, to contribute to disease phenotype, and to be therapy related predicting patient survival. Furthermore, just recently, DNA methylation and expression signatures have improved the classification of human DLBCLs (25). The method of bisulfite conversion of DNA is the most efficient to study methylation and is based on the selective chemical conversion of all the unmethylated cytosines into uracil by treatment with sodium bisulfite, which is then amplified as thymine during PCR. In contrast, the methylated cytosines are not converted, such that, in the final sequencing result, the 5-methylcytosine will be still detected as cytosine. Following these steps, various methods have been determined to quantify DNA methylation such as PCR, gel electrophoresis, southern blotting, mass spectrometry, and pyrosequencing and applied for specific loci or low throughput methylation analysis (26).

In the canine species, the effects of methylation have been scarcely investigated, especially in cancer. Few studies are reported that investigate perturbations of DNA methylation at single gene level, such as DLC1, ABCB1, and FHIT (27–29). DAPK, a suppressor of the cellular transformation at early stages of tumor development was found to be hypermethylated with high frequency in canine nodal high-grade B-cell lymphomas (30). DAPK acts as a tumor suppressor by sensitizing cells to many apoptotic signals, and the promoter methylation may influence the expression of this gene. In a different study, p16 hypermethylation and consequent silencing was demonstrated in few lymphoma cell lines by bisulfite sequencing method and by the indirect effect of a specific methylation inhibitor (31). Only one study was performed in canine DLBCL, and the bisulfite conversion followed by 454 amplicon sequencing approach was used for the first time in veterinary medicine. This approach appears to be the most innovative and, in this study, provided an accurate quantification of TFPI-2 methylation levels with a single base resolution, allowing the identification of CpG sites aberrantly methylated in canine DLBCLs. The selection of the gene was made on the previous identification of a significant downregulation of TFPI-2 mRNA levels in dogs affected by DLBCL compared with healthy lymph node controls (32). In future, a genome-wide approach to investigate global perturbations of methylation in canine lymphomas will become worthwhile to test the hypothesis that hypomethylation is able to increase genomic instability and to activate proto-oncogenes, whereas hypermethylation contributes to tumorigenesis by silencing tumor suppressor genes. The most promising methodologies are now based on high-throughput sequencing.

## Conclusion

In conclusion, most of the data reported in this review indicate the extreme heterogeneity of canine DLBCL and the lack of definitive diagnostic and therapeutic protocols. Several different approaches are currently in use, but are not comparable. This deficit affects both clinicopathological and experimental studies, including clinical trials. In this contest, the biological data that have been published in canine DLBCL are probably unique in veterinary oncology and are one of the most significative examples of the extreme biological diversity within a single tumor histotype. Canine DLBCL cannot be longer regarded as a single disease, because the biological behavior has been demonstrated to be dependent on its activated B-cell origin or not, association with mitotic index, blood and BM tumor infiltration, and specific genomic aberrations. In the future, therapeutic decisions will increasingly be correlated to the analysis of these data, and their significance need to be further validated in a larger cohort of dogs.

## Author Contributions

LA was the sole author who participated in drafting the review.

## Conflict of Interest Statement

The author declares that the research was conducted in the absence of any commercial or financial relationships that could be construed as a potential conflict of interest.
